# Influence of coal cowl parameters on the coal loading process of thin coal seam shearer drum

**DOI:** 10.1038/s41598-024-57372-9

**Published:** 2024-03-25

**Authors:** Weipeng Xu, Xiaodi Zhang, Kuidong Gao, Shenghao Ma

**Affiliations:** 1 College of Mechanical and Electronic Engineering, Shandong University of Science and Technology, Qingdao, 266590 Shandong Province People’s Republic of China; 2 China Coal Technology and Engineering Group, Shanghai Co., Ltd., Shanghai, 200030 People’s Republic of China; 3 School of Mechatronic Engineering, China University of Mining and Technology, Xuzhou, 221116 Jiangsu Province People’s Republic of China

**Keywords:** Thin coal seam shearer, Coal cowl, Loading performance, Particle’s dynamic behavior, Discrete element method, Engineering, Mechanical engineering

## Abstract

The low loading rate of the thin coal seam shearer drum is a severe obstacle to the efficient mining of thin seam resources, and the auxiliary drum loading through the cowl is an effective measure to alleviate this situation. However, the working mechanism of the coal cowl still remains unclear. In this paper, with the help of the discrete element method and the modeling experiment method, the effects of coal cowl’s offset distance, tilt angle and wrap angle on the coal loading rate under different loading modes of the drum are investigated; and the significance of various factors and their interactions to the drum coal loading rate is explored by designing response surface experiments. The findings show that a monotonous negative correlation between the offset distance of the coal cowl and the coal loading rate is identified, and that a smaller offset distance can effectively improve the coal loading rate of the drum. The conveying torque is significantly increased, easily inducing the drum choking, coal recycling coal over-crushing. Along with the increasing tilt angle, the rate of ejection loading decreases monotonically, and the rate of pushing loading increases first and then decreases. Coal loading rate is weakly affected by changes in coal cowl’s wrap angle. The results of response surface analysis reveal that the most significant factors affecting the drum’s coal loading rate are tilt angle and offset distance in ejection and pushing loading modes, respectively. The conclusions drawn here offer implications for improving the coal loading performance of the thin coal seam shearer drum, as well as certain guidance on the optimal design of coal cowl parameters.

## Introduction

With large proven reserves, the widely-distributed thin coal seams have been considered as important mining objects in mining areas. The advancement of mining technologies also contributes to higher yield of thin coal seam. However, due to the complicated mining environment, poor loading performance of the thin seam shearer drum has not been well solved; relying on manpower to clean up the floating coal not only causes a production safety hazard, but also reduces the productivity of the working face and affects the safe and efficient mining of coal.

In the early 1980s and 1990s, both the coal cutting performance and the effects of structural features on coal loading performance were comprehensively considered in the design of the shear drum. Hurt^1^ pointed out that the coal loading performance was affected by the number of vanes, the helical angle and depth of vanes, and the size of coal discharge port. Realizing that the shape of hub also exerts a significant impact on coal loading performance, Ayhan^2,3^ proposed a drum with a novel hub called, whose loading performance was improved to some extent compared to the drum with cylinder hub during in-situ test. Gao et al.^4–7^ investigated the rotational speed threshold of drum choking, as well as the regularity and significance of the influence of drum structure and motion parameters on the coal loading rate, demonstrating that the drum’s cutting depth and hauling speed had a significant impact on loading rate. Wydro^8^ discussed the effect of material filling rate on the loading performance by building a loading test bed, and provided a basis for matching between rotational speed and hauling speed of the drum. Zhang^9^ experimentally explored the effect of coal capacity space in the drum envelope zone on loading performance, and obtained the effect of the ratio of drum hub to vane diameter on coal loading rate. Liu et al.^10^ fully considered the influence of multi-parameter matching relationship between the structure and rotation speed of the drum and the haulage speed of the shearer on coal loading performance. By leveraging Taguchi’s orthogonal experiment, the significance of each factor on the coal loading performance was investigated, and the parameter combination relationship for the optimal coal loading performance was obtained.

The field test and scaled testing bed can reproduce how the coal shearer drum works in real life to the greatest extent, but involve high experimental cost and long duration. With the development of computer simulation technologies, numerical simulation method can be adopted for solving these issues. Working well in simulating the dynamic characteristics of particles, the discrete element method (DEM) proposed by Cundall^11^ works well in simulating has been widely applied in the field of granularity conveying and mixing^12–14^. Moreover, the mechanical and crushing behaviors of continuum, such as coal and rock^15,16^, can also be simulated by creating bonds between discrete particles, serving as a common practice in coal breaking by shearer drums and rock breaking by mechanical tools^17,18^. Coal particles are peeled off from the coal wall by the conical pick and transported to the scraper under the action of the spiral blade. This process is simulated and reproduced by Gao et al.^19^ with the help of DEM. Meanwhile, he further studied the influence of the position relationship of the three machines in the fully mechanized mining face on the coal loading performance of the drum. Subsequently, Tian et al.^20^ numerically probed into the influence of the factors such as the helical angle, rotation speed, haulage speed and cutting depth of the drum on the coal loading rate through DEM, and compared the simulation results with the in-situ test results, further confirming the effectiveness of DEM for the study of the drum’s loading performance. Zhao et al.^21^ numerically investigated the coal loading performance of the drum under the influence of multiple factors, and proved that the coal loading rate was influenced most by the cutting depth and least by the helical angle. Furthermore, they also provided the optimized structural and operational parameters of the drum. By resorting to DEM-based numerical simulation, not only the macroscopic results of drum loading rate can be obtained, but also the mechanism of drum coal loading can be revealed through the analyses of the particle motion trajectory, the particle micro contact characteristics, and the velocity field distribution characteristics. Based on this, Gao et al.^22^ developed a drum with a variable cross-section hub to improve the coal loading performance of the drum and studied the motion behavior of particles and the filling rate of the envelope zone to further identify the influence mechanism of the hub on the coal loading, thus obtaining the optimal shape and parameters. Moreover, by dividing the coal wall into different areas according to different cutting depths in the simulation, Gao et al.^23^ found that the broken coal particles at the larger cutting depth, more specifically, at the endplate position, are difficult to be conveyed to the scraper due to the long distance, resulting in the prevalence of scattered coal in the channel and the emergence of floating coal. To this end, they proposed a novel segmented differential drum in which the lower speed of the front drum reduces the tangential ejection speed of coal particles to lower the probability of being thrown into the goaf, and the higher speed of the rear drum helps to improve the loading speed of coal particles at the coal discharge port of the drum. The results obtained by Gao et al.^23^ show that the novel drum can improve the loading performance significantly, thus providing guidance on drum design. By utilizing the DEM to study the accumulation and movement of particles under the action of drum vanes, an axially inclined helical vane drum was designed by Sun et al.^24^, which could effectively improve the coal loading rate. In addition to figuring out the impact of the drum structure parameters on the coal loading performance through the above DEM-based numerical simulation, Gao et al.^25^ also further studied the conditions of the coal seam, i.e., the effect of the working face’s coal seam inclination angle and strike inclination angle on the loading performance, which provides guidance for selecting the appropriate mining loading strategy based on the parameters of different coal seams.

The shearer drum loads the falling coal into the scraper following the conveying principle of Archimedes spiral. Due to the working mode and installation structure characteristics of the drum, the conveying space is not closed during the loading of the falling coal, so it inevitably causes the accumulation of floating coal in the drum channel. Although the scholars in the above literature have adopted the improved structural and operating parameters of the drum and the shearer to improve the coal loading rate, as shown in Table 1, the coal loading performance of the drum does not improve too much, and the problem of poor coal loading performance cannot be effectively solved.

**Table 1:** Research status of coal loading performance of the drum.

Method	Factors	Main contents	Sources
In-situ test	Structural parameter	Globoid hub	3
Theoretical and laboratory investigation	Operational parameter	Space capacity model; Minimum rotation speed for avoiding drum clogging; Loading mode	4
Laboratory investigation	Structural and operational parameter	Hub diameter; cutting depth; vane helix angle; drum rotation speed; hauling speed;	5–7
Laboratory investigation	Filling rate	Filling rate	8
Laboratory and numerical investigation	Structural and operational parameter	Hub diameter; loading mode	9
Laboratory investigation	Structural and operational parameter	Vanes helical angle; drum rotation speed; haulage speed	10
DEM simulation	Arranging and connected equipment structural parameter	Relative height and distance between ranging arm and scraper; thickness of ranging arm	19
DEM simulation and in-situ test	Structural and operational parameter	Vanes helical angle; rotation speed; haulage speed; cutting depth	20,21
DEM simulation	Structural parameter and novel drum	Cross-section shape of hub; rotation speed matching; vanes helical angle	22,23
DEM simulation	Structural parameter	Axial tilt angle of helical vanes	24
DEM simulation	Geology parameter	Dip angle of work face; loading mode	25
In-situ test and DEM simulation	Novel shearer	Loader shape; installation angle;	26,27
DEM simulation	With or without cowl	Comparative of loading performance	33

**Figure 1:**
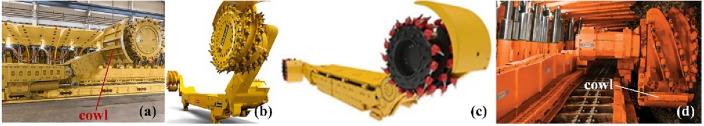
Coal shearer manufacturer's cowl products (**a** CAT; **b** Eickhoff; **c** FAMUR; **d** JOY)^28–31^.

For this reason, scholars have tried to enhance the coal loading rate of the drum with the aid of various devices. Boloz et al.^26^ improved the structure of the shearer, designed a new type of bladeless drum relying on the shovel plate to load the shearer prototype for mining thin and hard coal seams, and confirmed that the designed new shearer could significantly increase the daily output. Going on with this work, Zhang et al.^27^ simulated the cutting and loading process of the above new shearer by means of DEM, and dissected the influence of the structural form and key parameters of the shovel plate on the coal loading performance. Working differently from the traditional double drum shearer, the new shearer only relies on the drum to break coal, and loads the floating coal through the shovel plate. However, despite of having been proved to produce better coal loading effect, the new shearer cannot be easily popularized and applied on a wide scale at present. In contrast, the prevailing double drum shearer is generally installed with a cowl to form a closed conveying space for the drum to improve the loading rate^28^, as often seen in the products of major coal machine manufacturers in Fig. 1^29–32^. In addition, Gospodarczyk et al.^33^ verified via DEM that adding a cowl to the drum could effectively improve the coal loading rate when cutting the floor coal. However, only qualitative evidence on the improvement of drum’s coal loading performance assisted by cowl is obtained at present, and the underlying improvement mechanism still remains unclear. Due to many structural and installation parameters of cowl involved, greater efforts shall be made to figure out the influence of key parameters on drum’s coal loading performance.

Based on the above research, the thin seam shearer drum cowl is taken as the research object in this paper. With the help of DEM, dynamic characteristics of the particles in the enclosed space formed between the cowl and the coal wall are analyzed to further reveal auxiliary loading mechanism of the cowl. Based on the macro coal loading rate results, the influence of key parameters of the cowl on the coal loading performance of the drum is clarified, thereby providing certain guidance on the structural design and installation parameters determination of the coal cowl. The main structure of this paper is arranged as follows: Firstly, the research status of the loading performance of thin coal seam shearers is summarized; Secondly, a numerical model of drum loading process is constructed using DEM, and the effectiveness of the established numerical model is verified by comparing it with the simulation-based experimental results; Then, a single factor exploration is conducted on the coal cowl’s key parameters that affect the coal loading performance; Finally, the response surface optimization method is used for exploring the significance of the impact of various key parameters on the coal loading rate, and the optimal matching relationship of key parameters is acquired.

## Simulation modeling

### Discrete element particle contact model

**Figure 2:**
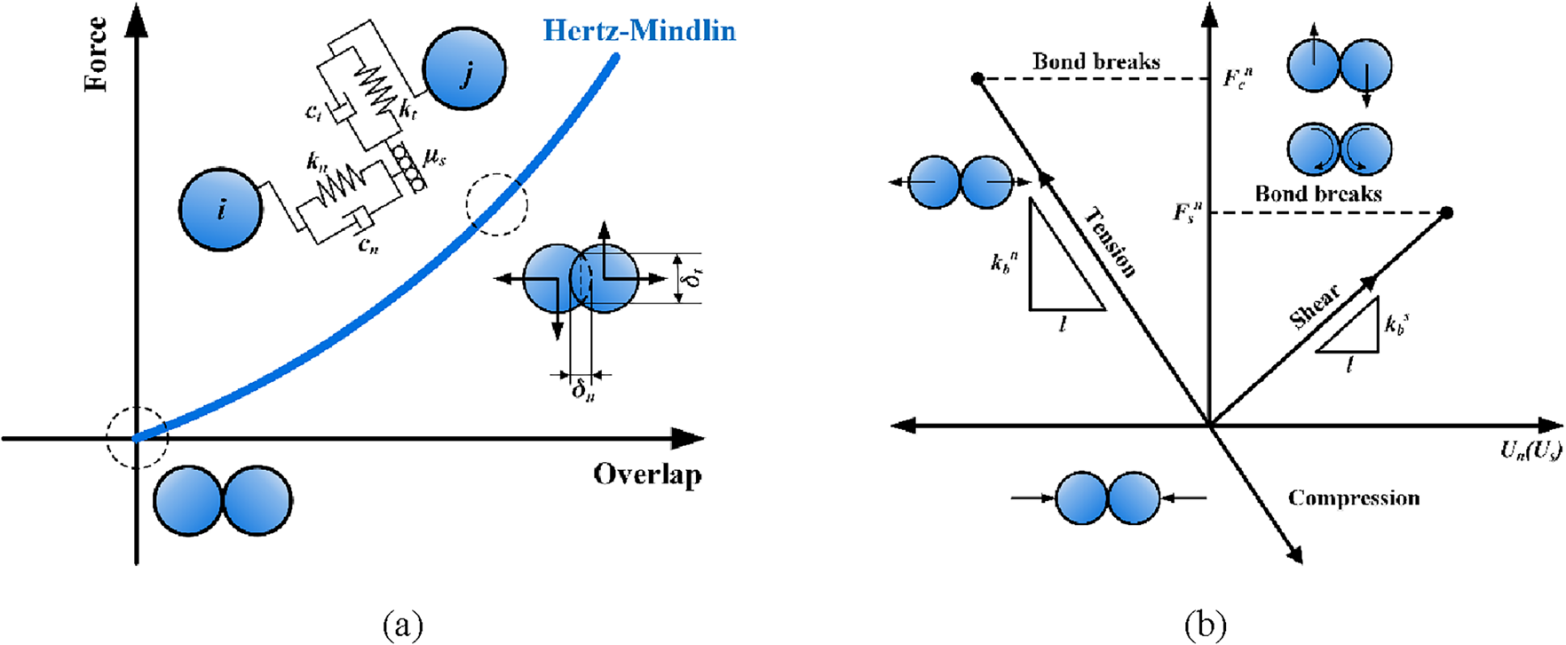
Schematic diagrams of particle contact models; (**a**) Hertz–Mindlin linear contact model; (**b**) Bonded particle contact model.

Cundall^11^ proposed the DEM to simulate the mutual contact characteristics between discrete particles, with the fundamental contact model of particles being a mechanical system composed of spring and damp to characterize the normal and tangential contact of particles. Noticeably, the Hertz-Mindlin model was also widely employed in the previous studies^22–24^, as shown in Fig. 2a, whose mechanical expression is given by Eq. (1):

1 $$\left\{ {\begin{array}{*{20}l} {F_{n} = \frac{4}{3}E^{*} \sqrt {R^{*} } \delta_{n}^{\frac{3}{2}} } \hfill \\ {F_{t} = - S_{t} \delta_{t} } \hfill \\ \end{array} } \right.$$

wherein *F*_*n*_ is the normal force, N; *δ*_*n*_ is the normal overlap between the particles, mm; E^*^ is the equivalent Young’s modulus, GPa; R^*^ is the equivalent radius of the particles, mm; *F*_*t*_ is the tangential force, N; *S*_*t*_ is the tangential stiffness of the particles, N/m; *δ*_*t*_ is the tangential overlap between the particles, mm.

Since the working process of the coal miner drum involves cutting and loading coal, the bonding model^34^ is used as an additional contact model to bond the discrete particles into a simulated coal wall as a whole. The principle of the bonding model is to establish a virtual bonding between discrete particles, thus resisting tensile and shear damages. When the external load exceeds the threshold of its tensile or shear strength, the microscopic bond breaks, as manifested by the crushing and spalling of the coal block in Fig. 2b. The bonding model can be expressed as:

2 $$\left\{ {\begin{array}{*{20}l} {\delta_{{F_{n} }} = - \pi R_{B}^{2} V_{n} S_{n} \Delta t} \hfill \\ {\delta_{{F_{t} }} = - \pi R_{B}^{2} V_{t} S_{t} \Delta t} \hfill \\ {\delta_{{M_{n} }} = - \frac{1}{2}\pi R_{B}^{4} \omega_{n} S_{t} \Delta t} \hfill \\ {\delta_{{M_{t} }} = - \frac{1}{4}\pi R_{B}^{4} \omega_{t} S_{n} \Delta t} \hfill \\ \end{array} } \right.$$

wherein *M*_*n*_ and *M*_*t*_ are the torques in normal and tangential directions of bond key, N·m, respectively; *R*_*B*_ is the bonding radius, mm; Δ*t* is the time step, s; *S*_*n*_ and *S*_*t*_ are the normal and tangential stiffnesses, N/m, respectively; *V*_*n*_ and *V*_*t*_ are the normal and tangential velocities of the particles, m/s, respectively; and *ω*_*n*_ and *ω*_*t*_ are the normal and tangential angular velocities, rad/s, respectively.

The normal *σ*_max_ and tangential *τ*_max_ fracture thresholds for bonds are given by Eq. (3):

3 $$\left\{ {\begin{array}{*{20}l} {\sigma_{\max } < \frac{{ - F_{n} }}{{\pi R_{B}^{2} }} + \frac{{4M_{t} }}{{\pi R_{B}^{4} }}R_{B} } \hfill \\ {\tau_{\max } < \frac{{ - F_{t} }}{{\pi R_{B}^{2} }} + \frac{{2M_{n} }}{{\pi R_{B}^{4} }}R_{B} } \hfill \\ \end{array} } \right.$$

### DEM modeling of coal loading in cowl

**Figure 3:**
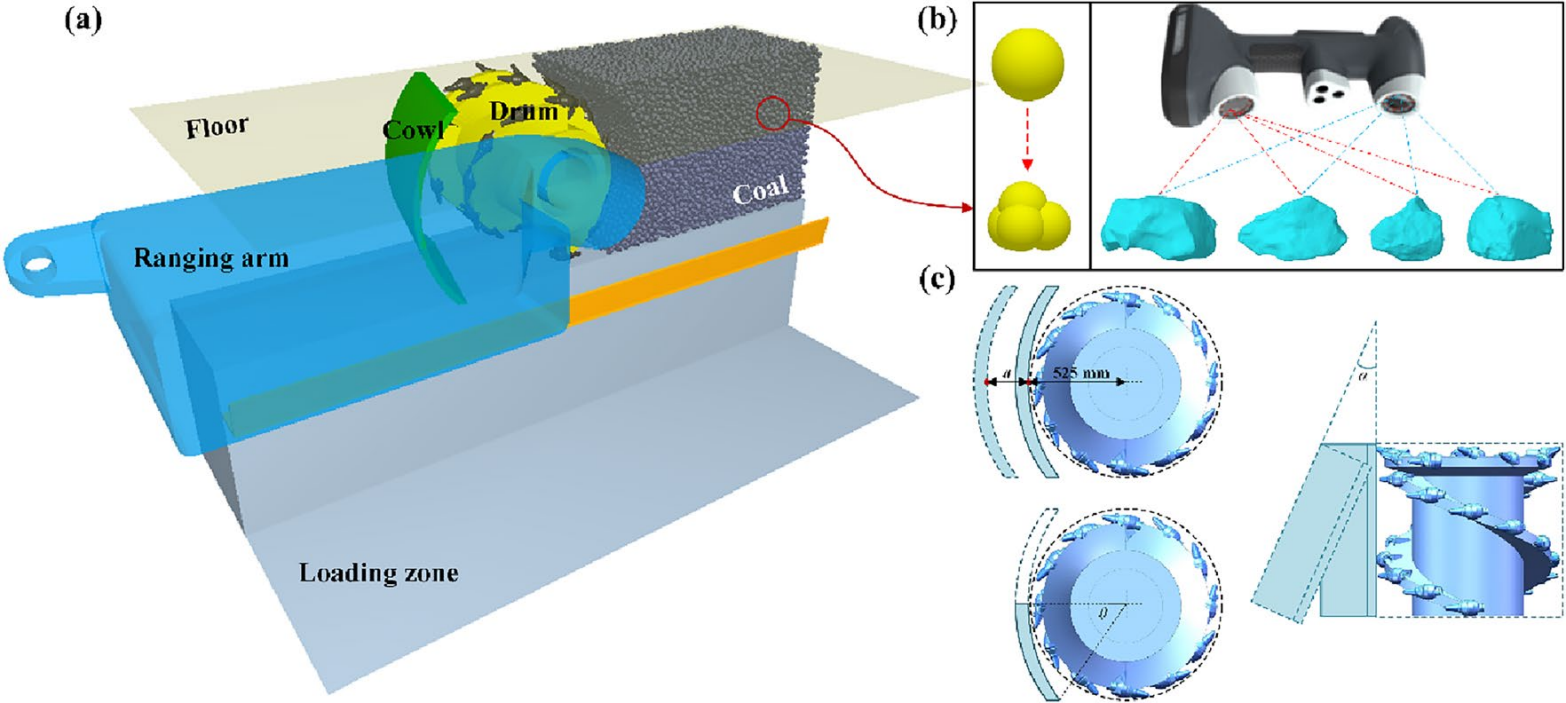
DEM-based numerical modeling; (**a**) numerical modeling of coal loading by cowl-assisted drum; (**b**) particle modeling in simulation; (**c**) schematic diagram of key parameters of the cowl.

In the working process of thin coal seam shearer, the coal loading rate is not only affected by the restricted and narrow space in the working face, but also significantly subjected to the rocker arm of the coal shearer and the arrangement parameters between the three machines^19^. Therefore, when constructing the numerical model of coal cowl-assisted drum cutting and loading, it is necessary to establish the top and bottom plate wall, drum and coal cowl models, as well as structural models of rocker arms and scraper shovels, etc. Due to the small thickness of the thin seam mining, the diameter of the drums is approximately the same as the thickness of the seam. The mining strategy of the commonly used double-drum thin coal seam shearer is to cut the coal in the front drum and clean the floating coal in the rear drum. Therefore, only the front drum and rocker arm are modeled in the simulation. In the meantime, in order to improve the simulation efficiency and shorten the calculation time, the connection mechanism and hydraulic drive mechanism, which do not have an obvious influence on the coal loading results, are omitted, and the numerical model constructed is shown in Fig. 3a.

In DEM simulation, the shape of particles exerts a significant effect on the simulation results^9,35–37^. Coal, as a kind of typical non-homogeneous material with random shape and size, breaks and crumbles under the cutting action of the drum trunnion, making it difficult to comprehensively and accurately characterize its external morphology in DEM simulation. Yet, scholars like Liu et al.^38^ found that the particle size of the broken coal by the drum trunnion obeys a certain distribution and exhibits fractal characteristics through experimental research. Also, Yang et al.^39^ constructed 3D cloud maps of 135 typical coal particles with a 3D laser scanner, and provided a corresponding database. Therefore, combining the existing data and literature results^7^, the four-sphere filling model is selected for modeling the simulated particle shape in this paper, as shown in Fig. 3b. In addition, since this paper focuses on the coal loading performance of drum, given that the strength factor has no significant impact on drum conveying and loading, it is only necessary to ensure that the coal wall does not collapse in the process of drum coal cutting when determining the particle bonding parameters. Meanwhile, the physical parameters (including restitution coefficient, sliding friction, and rolling friction) in DEM that have a significant impact on particle conveying and loading performance, were calibrated by rebound test, sliding test, and angle of repose test respectively in literature^9,24^, and the calibrated parameters are then adopted in this paper. The physical and bonding parameters of the particles in DEM and the geometric model parameters are shown in Tables 2 and 3, respectively. The influencing mechanisms of the installation distance, tilt angle and wrap angle of the cowl on coal loading rate of the drum are investigated, as shown in Fig. 3c. The range of key parameters for each factor studied in the simulation is presented in Table 4.

**Table 2:** Physical and bonding parameters of particles in simulation.

Parameter	Value
Particle structure	
Particle shape	Pyramidoid (four-particle filled)
Equivalent radius/mm	15
Particle physic parameter	
Density/(kg/m^3^)	1280
Poisson’s ratio	0.28
Young’s Modulus/GPa	4.25
Particle–particle contact parameter	
Restitution coefficient	0.25
Sliding friction	0.80
Rolling friction	0.20
Particle–wall contact parameter	
Restitution coefficient	0.40
Sliding friction	0.05
Rolling friction	0.50
Bonded parameters	
Normal stiffness/(N/m^3^)	9.50e9
Shear stiffness/(N/m^3^)	5.50e9
Critical normal stress/pa	5.56e6
Critical shear stress/pa	1.20e6

**Table 3:** Structural and motion parameters of drum in simulation.

Parameter	Value
Structural parameters	
Cutting diameter of picks/mm	1050
Diameter of helical vanes/mm	950
Diameter of barrel/mm	550
Drum width/mm	850
Number of helical vanes	2
Helical angle of vanes/°	21
Motion parameters	
Rotation speed/rpm	60
Hauling speed/(m/min)	6.0

**Table 4:** Key structural parameters of the cowl.

Parameter	Value
Offset distance/mm	0, 75, 150, 225, 300, 375, 450
Tilt angle/°	0, 5, 10,15, 20, 25, 30
Wrap angle/°	30, 45, 60, 90

### Validation of the simulation model

**Figure 4:**
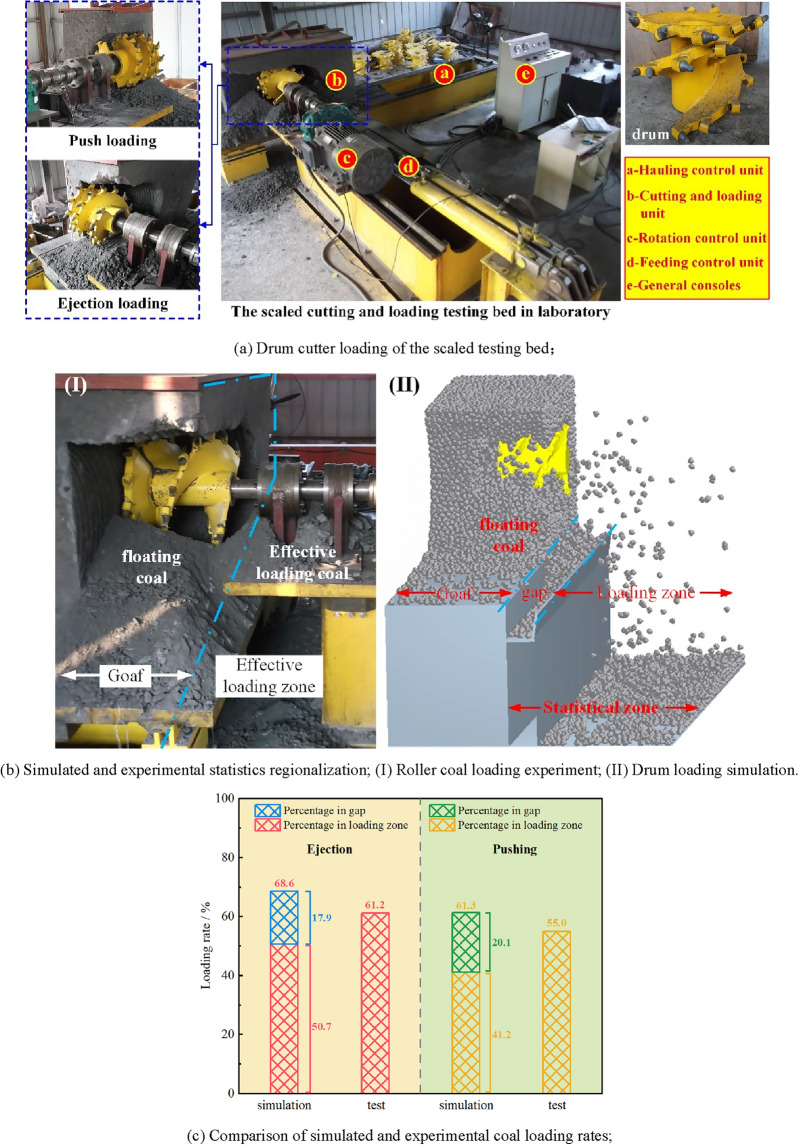
Comparison of experimental and simulated drum coal loading results.

To confirm the accuracy of the simulation results under the selected particle parameters, the established numerical model is validated by means of a scaled testing bed, which mainly consists of a test drum, an artificial coal wall, a hauling unit, a rotational speed unit, and a total control unit, as shown in Fig. 4a. Specifically, the artificial coal wall is composed of coal ash, cement, and water, among which the coal ash is used for revealing the mechanical properties of the real coal as much as possible and the cement improves the whole strength of the coal wall, with their proportion of mass given in Table 5. In order to avoid the influence of other factors on comparative differences between the simulation findings and the experimental testing results, the drum model in the validation simulation adopts the same structure and motion parameters as those of the scaled testing bed, as detailed in Table 6.

**Table 5:** Composition of artificial coal wall.

Material	Coal ash	Cement	Water
Proportion/%	70.0	12.0	18.0

**Table 6:** Drum structure and motion parameters in the validation experiment.

Parameter	Value
Loading mode	Pushing; ejection
Structural parameters	
Cutting diameter of picks/mm	530
Diameter of helical vanes/mm	450
Diameter of barrel/mm	240
Drum width/mm	450
Number of helical vanes	2
Helical angle of vanes/°	21
Motion parameters	
Rotation speed/rpm	45
Hauling speed/(m/min)	6.0

The loading results after simulation and experiment are shown in Fig. 4b. Due to the space and structure limitations of the experiment, the falling coal zones are divided into the goaf and the efficient loading zone according to its distribution. In contrast, by setting the scraper in the simulation, the falling coal zones can be divided into the goaf, the gap zone and the coal loading zone. And the drum loading rate is calculated based on the ratio of the mass of coal particles in the coal loading zone to the total falling coal mass. However, in order to ensure consistent statistical method of simulated and experimental loading rate, the sum of the masses of the particles in the gap zone and the coal loading zone is taken as the effective loading mass in the simulation, and the ratio of effective loading mass to the total mass of the cut and broken particles is seen as the effective loading rate of the drum. The comparative results of the experimental and simulated loading rates under the two cutting modes of ejection and pushing loading are offered in Fig. 4c. Through the simulation, the two loading modes are 7.4% and 6.3%, respectively, which further confirms the feasibility of the proposed numerical model and the accuracy of the simulation results.

## Results and discussions

As mentioned above, the installation parameters affecting the performance of cowl loading mainly include the offset distance from the drum, the tilt angle, and the wrap angle. Therefore, through DEM simulation, the single-factor influencing mechanism of the three factors on the coal loading rate of the drum is numerically investigated, so as to grasp the range of parameter values in the case of better coal loading rate under each factor.

### Influence of the distance between cowl and drum on the coal loading process

**Figure 5:**
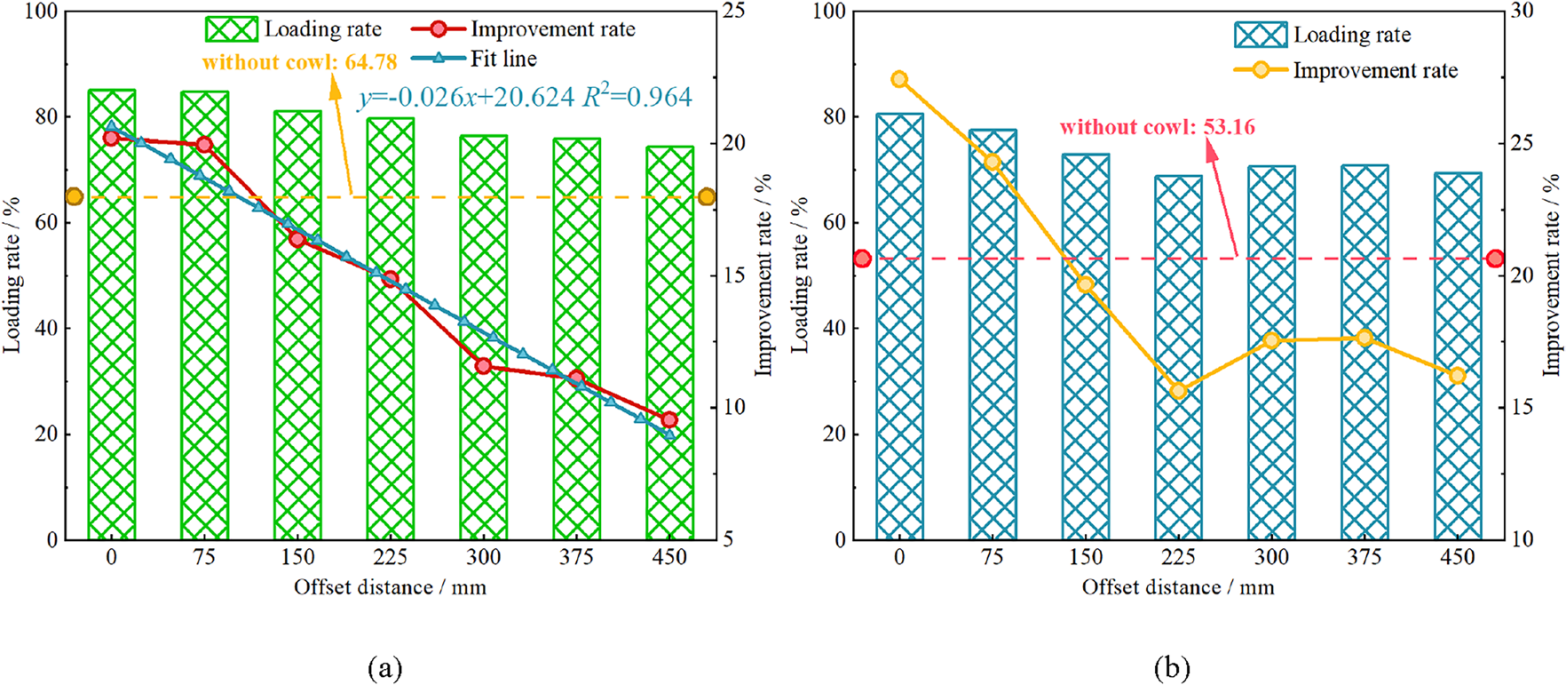
Relationship between the offset distance of the cowl and the coal loading rate; (**a**) Ejection loading; (**b**) Pushing loading.

**Figure 6:**
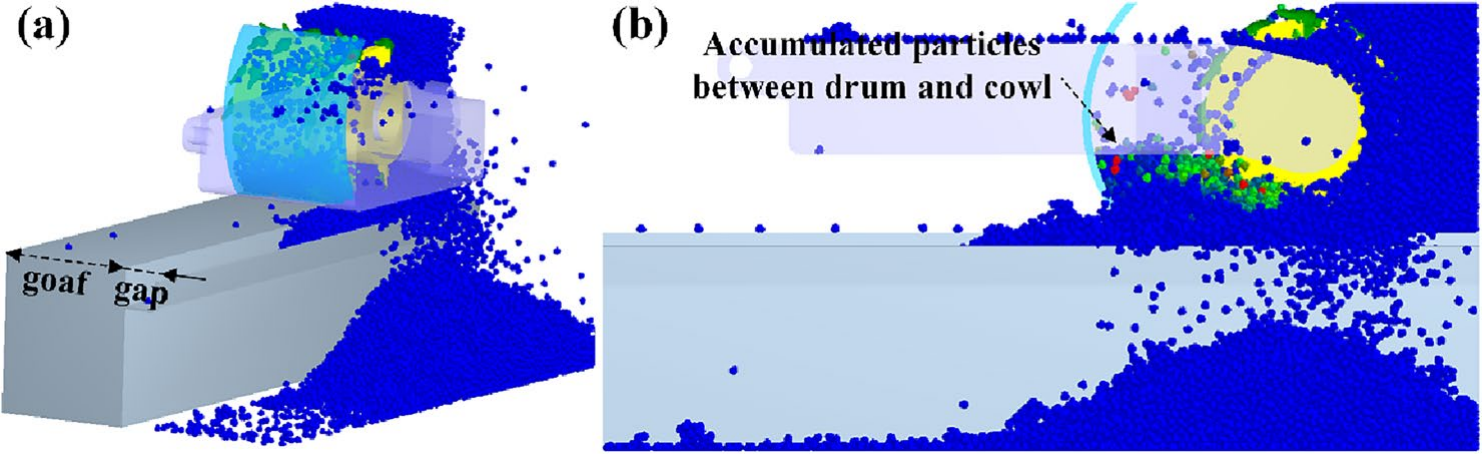
Simulation flash map during ejection loading; (**a**) *a* = 0 mm; (**b**) *a* = 450 mm.

Under the conditions of 0° tilt angle and 90° wrap angle, the changes of drum’s coal loading rate with offset distance under ejection and pushing loading conditions are obtained respectively, as shown in Fig. 5. Compared with the drum without the cowl, the cowl greatly enhances the drum’s coal loading rate in both loading modes. As seen in Fig. 5a, under the ejection loading condition, when the offset distance *a* = 0 mm is more than 20%, the drum’s coal loading rate enhances by 85%. The reason lies in that the cowl and the coal wall form a completely closed space, leaving no floating coals in the machine channel, but some particles are left in the gap zone and cannot be loaded, as shown in Fig. 6a. The lifting rate decreases approximately linearly with the increase of the offset distance; when the offset distance of the cowl reaches 450 mm, the lifting rate is still 10% compared with that of the no-cowl condition. as Accountably, along with the increasing offset distance of the cowl, the closed space formed between the cowl and the coal wall also gradually increases, and the envelope effect of the cowl on the drum decreases; part of the falling coal accumulated between the cowl and the drum cannot be loaded, as shown in Fig. 6b.

Due to different particle transport loading mechanisms of the drum under the ejection and pushing loading modes^7^, especially the front drum under the pushing mode, loading coal particles to the direction of the scraper will easily induce rocker arm choke. It can be seen from Fig. 5b that under the same offset distance of the coal cowl, the ejection loading rate is about 5% higher than the pushing loading rate. However, in case of the offset distance *a* = 0 mm, the drum pushing loading rate is still greater than 80%, enhanced by over 15% under the action of coal cowl, outperforming that of the drum under ejection loading, and proving that the enclosed space provided by the coal cowl for the drum is more beneficial to pushing loading.

**Figure 7:**
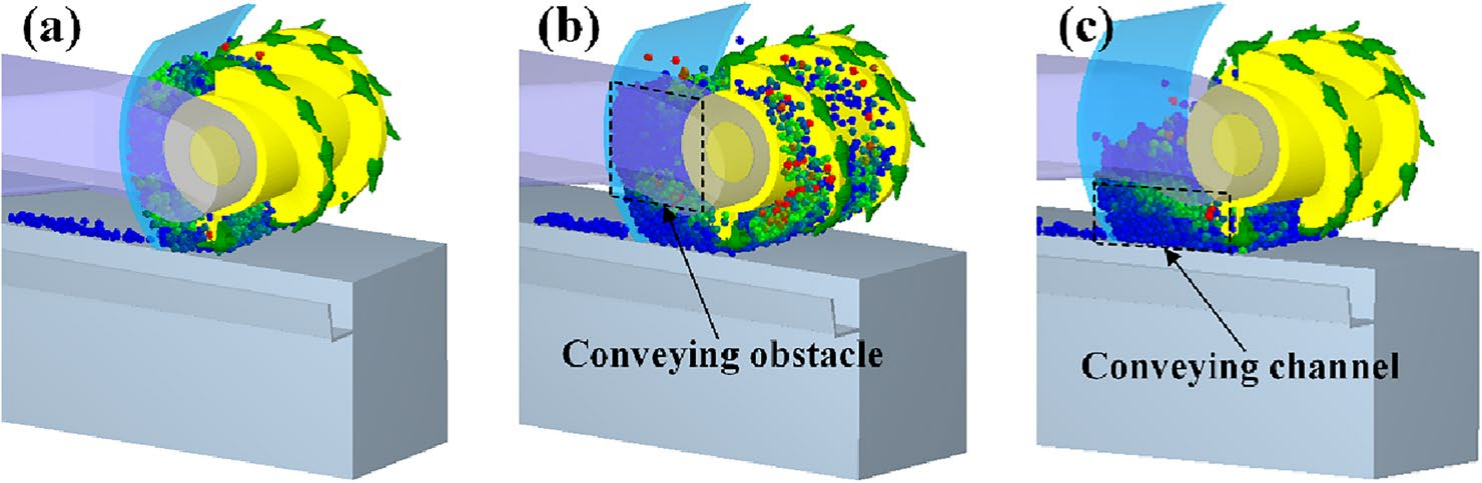
Simulation flash map during pushing loading; (**a**) *a* = 0 mm; (**b**) *a* = 225 mm; (**c**) *a* = 375 mm.

In addition, Fig. 5b demonstrates that under the condition of pushing loading, the enhancement of coal loading rate does not decrease monotonically with the increase of the offset distance of the coal cowl. The enhancement decreases approximately linearly and rapidly when the offset distance is increased from 0 to 225 mm, which is consistent with the results of ejection loading for the same reason. The enhancement increases slightly and then decreases again with the increase of the offset distance when the offset distance is more than 225 mm. This is mainly due to the different heights of particles accumulated between the coal cowl and the drum at different offset distances, as shown in Fig. 7. In case of the offset distance *a* = 225 mm, particles between the coal cowl and the drum are found with greater accumulation height, which is seriously choked by the rocker arm. With the increase of the offset distance of the coal cowl, the accumulation height of the particles between the coal cowl and the drum decreases, and the particles rely on their own flow properties to pass under the rocker arm and complete the loading, leading to a slight increase in the coal loading rate.

**Figure 8:**
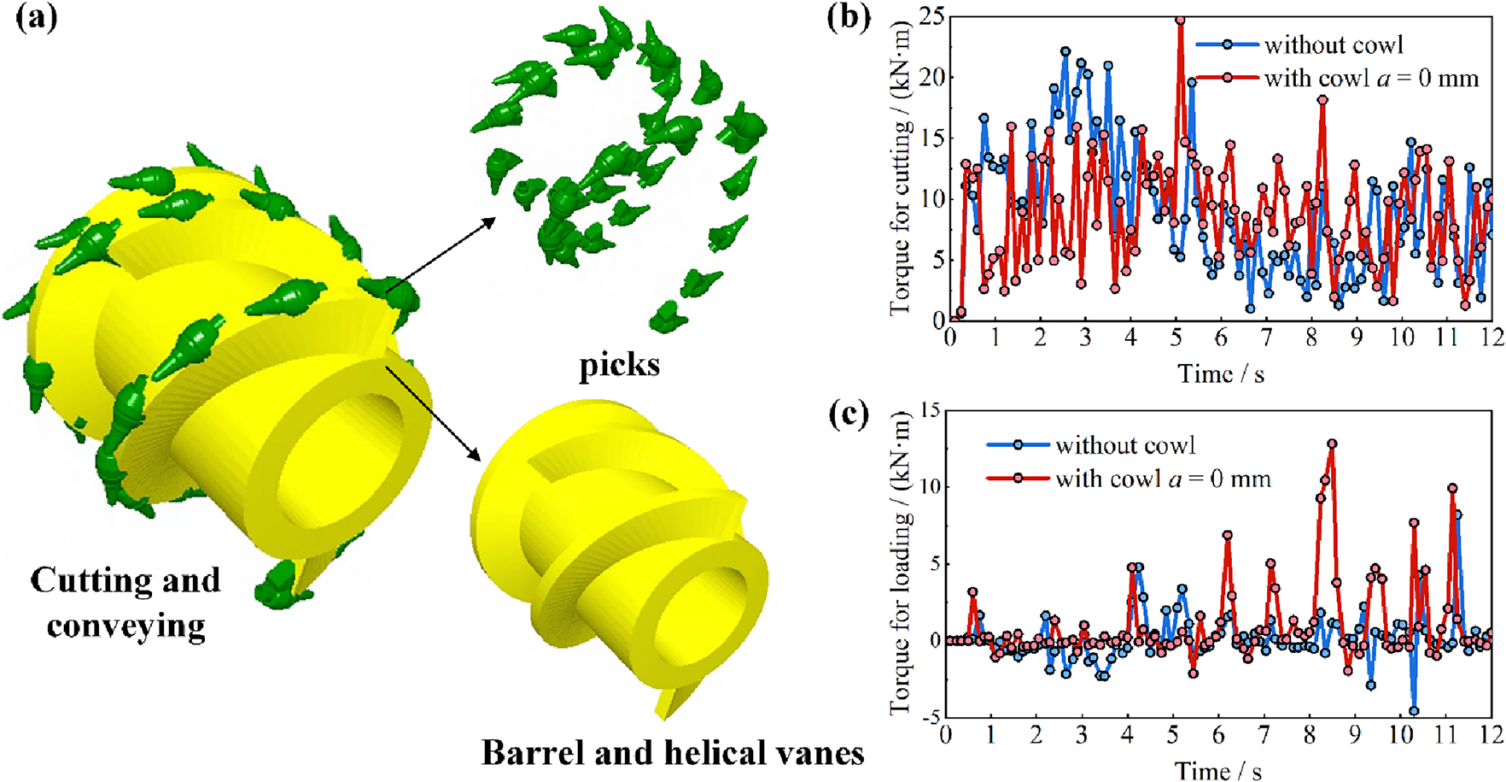
Separation treatment of drum cutoff and delivery torque; (**a**) model of cutoff and drum hub and vane separation; (**b**) comparison of time-course curves of cutoff torque; (**c**) comparison of time-course curves of delivery torque.

**Figure 9:**
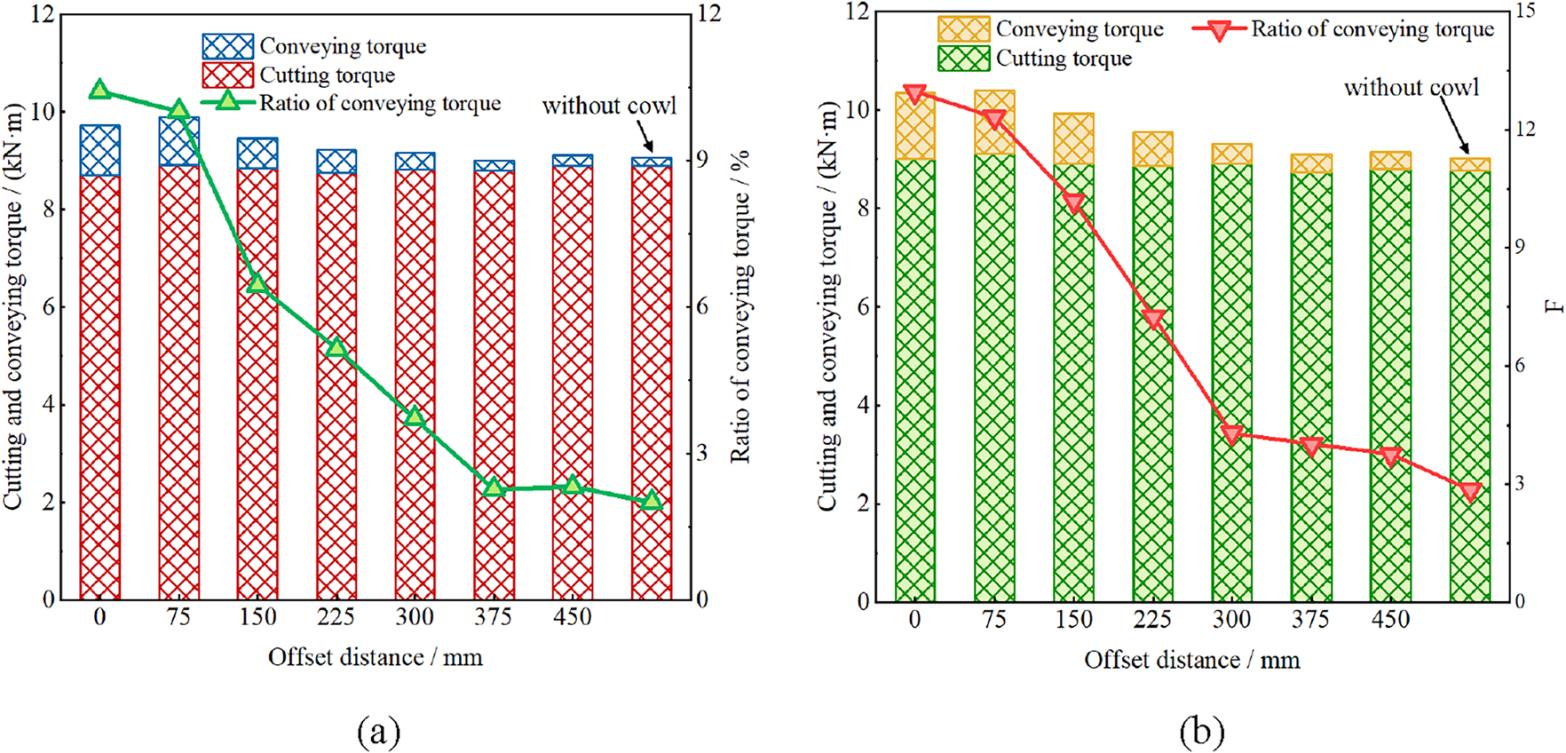
Effect of cowl’s offset distance on drum’s conveying torque; (**a**) ejection loading; (**b**) pushing loading.

Although a smaller offset distance of coal cowl greatly increases the drum’s loading rate, the drum being in a completely enclosed space formed by the coal cowl and the drum will obviously lead to an increase in the chances of recycling or over-crushing coal lumps, and drum choking problems. For this reason, by modeling the drum’s cutting mechanism (cutting teeth) and conveying mechanism (spiral vanes and drum hub) separately to calculate their working torques during the simulation process, it is indirectly predicted whether the drum will be choked or over-crushed, as shown in Fig. 8a. Taking the working conditions of offset distance *a* = 0 mm with or without coal cowl in the drum ejection mode as an example, the time-distance curves of the cutting and conveying torques are obtained as shown in Fig. 8b, c, respectively. Thus, it can be seen that under the conditions of offset distance of 0 mm with or without coal cowl, the cutting torque of the cutting teeth, i.e., the cutting torque of the drum, is characterized by periodic fluctuations; the fluctuation laws for both cutting and conveying torques are approximately the same, and the torque peaks do not vary significantly. This is mainly due to the fact that under the same simulation conditions, both the particle bonding parameters of coal wall and the cutting parameters of the drum are the same. Consequently, there is no significant difference in the cutting force and torque of the cutting teeth during the cutting process. However, seeing from the conveying torque in Fig. 8c, the conveying torque of the drum in the presence of coal cowl is obviously larger than that of the case without coal cowl, with the corresponding curve displaying obvious periodic peaks, and the peak value even close to the peak torque of the cutoff. Although the conveying torque curve for the case without coal cowl also exhibit fluctuating characteristics, the number of wave peaks and the peak value are significantly smaller than that of the case with coal cowl. This result indirectly indicates that the presence of coal cowl leads to the choking problem of the drum, and the drive motor needs to allocate more power consumption for driving the drum to convey the falling coal. In order to clarify the influence of the offset distance of the coal cowl on the conveying torque of the drum and select the offset distance parameter of the coal cowl, the average conveying torque of the drum is used as the evaluation index. Meanwhile, considering that the difference of the cut-off power of the drum under the same simulation parameter is not obvious, the conveying torque as a percentage of the *R*_*ct*_ is introduced on the basis of the average conveying torque, as given in Eq. (4). The influence of the coal cowl on the choking of the drum is further evaluated. The results of the corresponding drum’s conveying torque ratio at different offset distances are shown in Fig. 9.

4 $$R_{ct} = \frac{CT}{{CT + PT}}$$

wherein *CT* denotes the average conveying torque, kN m; *PT* represents the average cutting torque, kN m.

As can be seen from Fig. 9, in both ejection and pushing loading modes, the drum’s conveying torque ratio decreases monotonically with the increase of the offset distance of the cowl; especially, when the offset distance is less than 150 mm, the drum’s conveying torque ratio *R*_*ct*_ exceeds 10% and 12% in ejection and pushing loading modes, respectively. These results reveal that, on the one hand, the particle filling rate in the enclosed space is higher in case of a smaller offset distance of the cowl, on the other hand, the chances of recycle coal and plugging of the drum in the conveying process increase. In addition, by comparing the conveying torque ratios in these two modes, it can be seen that the conveying torque ratio in the pushing loading mode is significantly higher than that in the ejection loading mode, which is especially significant in the case where the offset distance of the cowl is less than 225 mm. In comparison with Fig. 7, it can be analyzed that the axial movement of the coal flow is seriously choked by the rocker arm during pushing loading, resulting in a larger conveying torque of the drum.

From the perspective of coal loading rate, the offset distance should be reduced as much as possible when designing and installing the coal cowl, so as to improve the drum’s loading rate. However, by dissecting the drum’s conveying torque under different offset distances, it is known that smaller offset distances of the coal cowl will lead to increased chances of drum choking, recycle coal and coal over-crushing. Therefore, seeing from Figs. 5 and 9 and considering the loading performance and conveying torque of the drum, the offset distance of coal cowl should be better within the range of 150–225 mm.

### Influence of the tilt angle of cowl on the coal loading process

**Figure 10:**
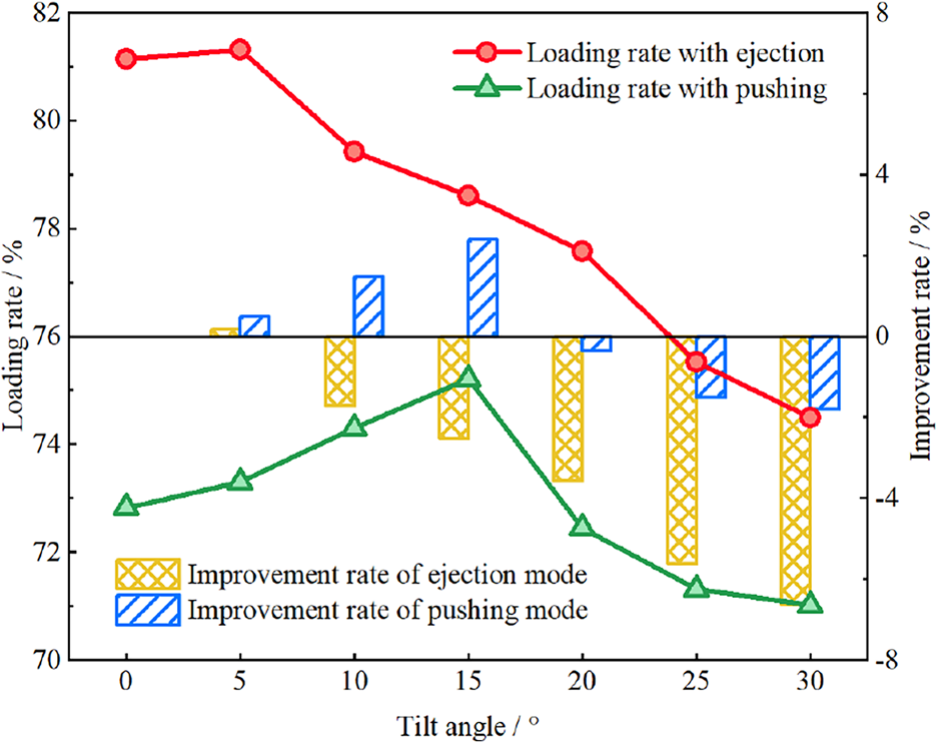
Relationship between cowl’s tilt angle and coal loading rate.

**Figure 11:**
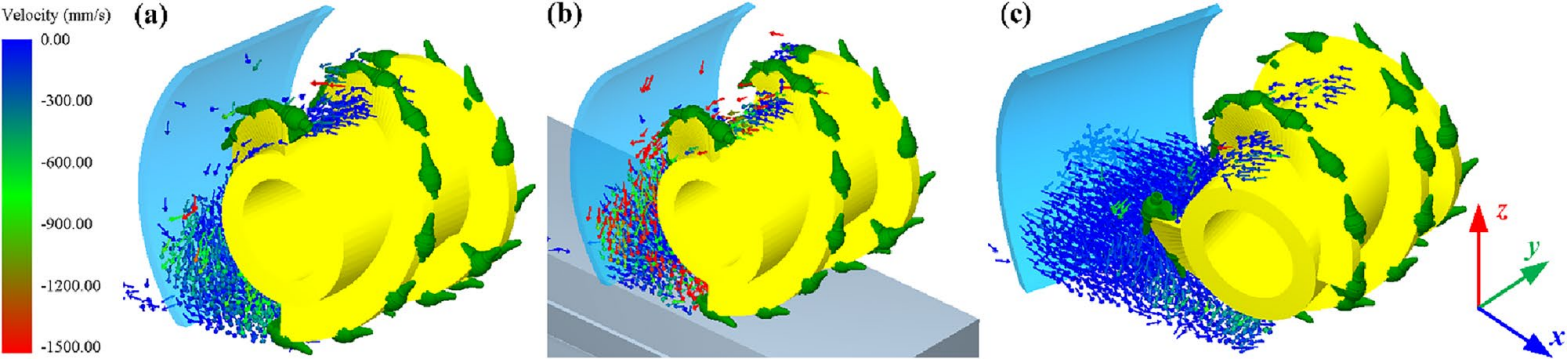
Particle velocity vectors of the cowl and drum in the envelope space under ejection loading conditions; (**a**) 0°; (**b**) 5°; (**c**) 30°.

As an important installation parameter of the coal cowl, the tilt angle also affects the coal loading rate of the drum. The influence of the tilt angle on the coal loading rate under the conditions of constant wrap angle *θ* = 90° and offset distance *a* = 150 mm is further investigated in the two modes of ejection and pushing loading, as detailed in Fig. 10. In the ejection loading mode the coal loading rate of the drum is obviously improved when the coal cowl’s tilt angle is 5°. In case that the tilt angle is greater than 5°, the increase of the coal cowl’s tilt angle is unfavorable to further improve the coal loading performance of the drum. When the coal cowl tilt angle is increased from 5° to 30°, the coal loading rate decreases by about 5%. The underlying reason lies in that the existence of the tilt angle increases the distance between the coal cowl and the end of the drum, leading to the accumulation of particles thrown to the goaf and making them impossible to be loaded, as shown in Fig. 11. Through the particle velocity vector diagram, it can be seen that, on the one hand, in case of a small tilt angle of coal cowl, the distance between the coal cowl and the end of the drum does not increase obviously, and the accumulated particles are more significantly affected by the spiral conveying action of the drum; on the other hand, the inclination of the coal cowl to the side of the effective loading zone has a positive effect on the loading of particles between the coal cowl and the drum. Thus, a secondary loading of particles occurs in the envelope zone, so the coal loading rate of the drum increases slightly relative to that of the non-tilted coal cowl. Meanwhile, the axial flow velocity of the particles in the envelope zone increases significantly under the effect of coal cowl tilting, as can be seen in Fig. 11. When the coal cowl’s tilt angle reaches 30°, the particles between the coal cowl and the drum in the envelope zone accumulate, with smaller influence of the drum spiral conveying effect and the coal cowl’s tilt angle, so the coal loading rate decreases more obviously.

**Figure 12:**
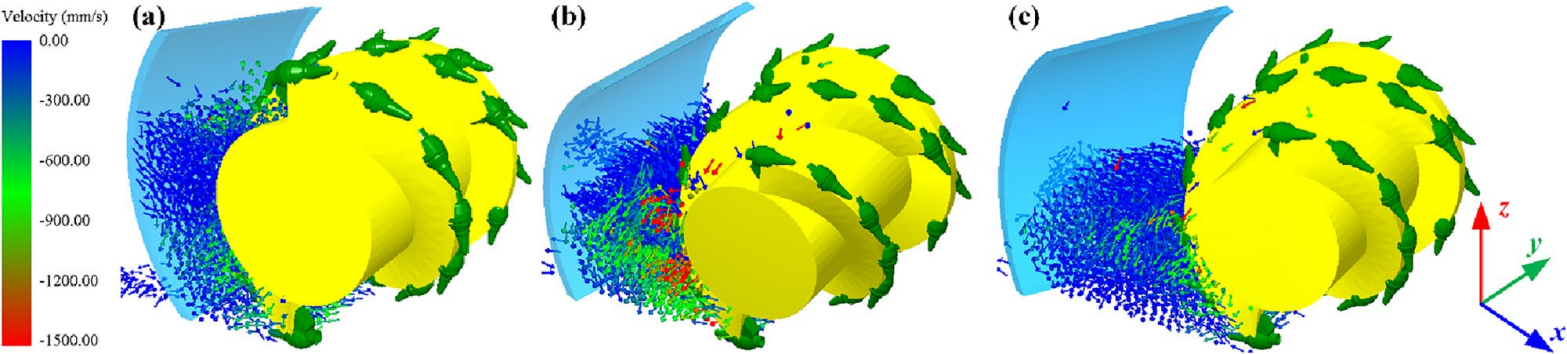
Particle velocity vectors of the cowl and drum in the envelope space under pushing loading conditions; (**a**) 0°; (**b**) 15°; (**c**) 30°.

The coal loading rate of the drum in the pushing loading mode shows an approximate quadratic relationship with the increase of the tilt angle, which increases monotonically with the tilt angle in the range of 5–15°, and then decreases gradually with the increase of the tilt angle. Therefore, a negative impact on the coal loading rate of the drum can be identified when the tilt angle exceeds 20°. Overall, the effect of coal cowl’s tilt angle on drum’s pushing coal loading rate is slightly smaller compared to that in the ejection loading mode. The velocity vector diagrams of the particles between the coal cowl and the drum in the envelope zone at different tilt angles of the coal cowl given in Fig. 12 show that when the tilt angle is 0°, the coal cowl provides a closed space for the drum so that the spiral vanes can continuously convey the coal and thus increase the coal loading rate (see Fig. 12a). With the increase of the tilt angle to 15° (see Fig. 12b), the coal cowl tilts to the coal loading zone, providing additional axial flow velocity for the particles in the envelope zone and thus increasing the coal loading rate. When the tilt angle continues to increase to 30°, the distance between the coal cowl and the end of the drum becomes larger, and the particles in the envelope zone are only close to the drum, which are significantly affected by the conveying effect of the spiral vane, as shown in Fig. 12c; due to the small axial flow velocity of the accumulated particles, the drum’s coal loading rate also decreases.

### Influence of the wrap angle of cowl on the coal loading process

Based on the above research, it is found that the coal cowl forms a closed space with the coal wall, which reduces the chance of coal particles being thrown to the goaf as floating coal due to the tangential movement of the drum, and thus improves the loading rate of the drum. In addition, according to the above analyses, the coal cowl plays a certain role in guiding the movement of the coal flow and improving its axial movement speed, further promoting the auxiliary drum loading. However, as can be seen in Figs. 7, 9, and 10, the particles are not completely filled between the drum and the coal cowl, so providing an incomplete wrap for the drum is sufficient to improve the coal loading rate by changing the wrap angle of the coal cowl plate. Reducing the wrap angle of the coal cowl plate not only saves the manufacturing cost of the coal cowl plate structure, but also improves the flexibility of the coal cowl plate to make attitude adjustments within a narrow operating space.

**Figure 13:**
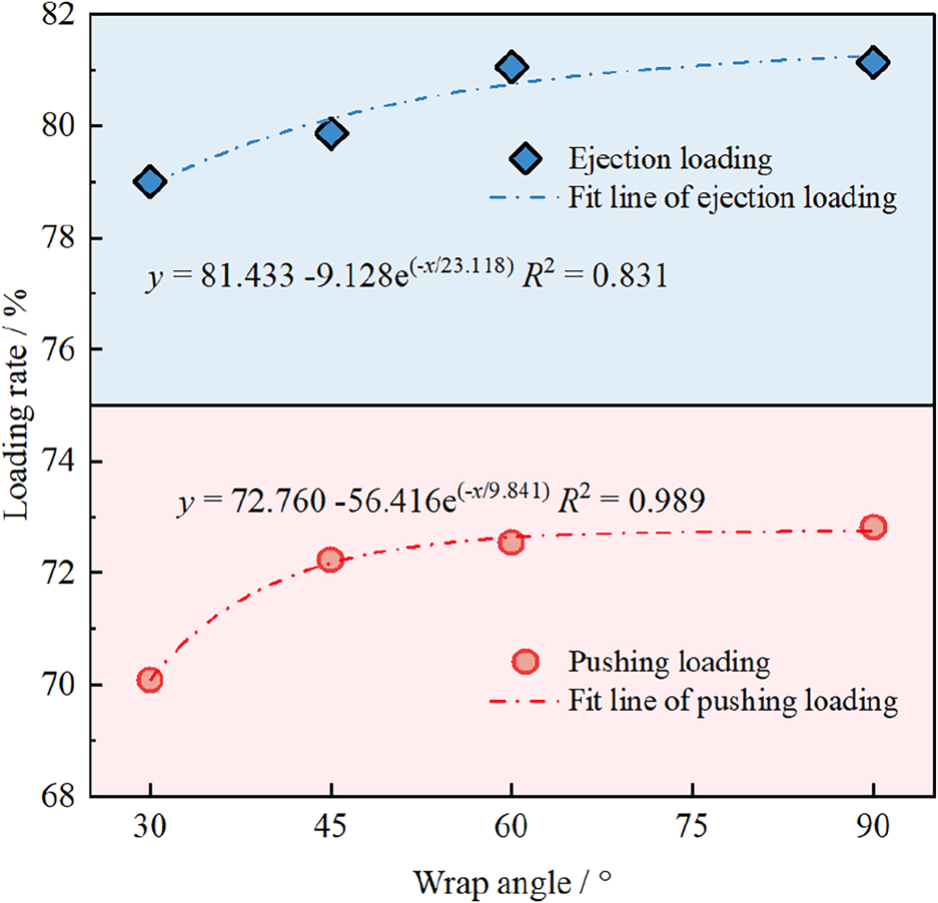
Relationship between coal loading rate and wrap angle under ejection loading and pushing loading modes.

Under the conditions of constant offset distance *a* = 150 mm and tilt angle *α* = 0°, the coal cowl’s wrap angle of 30°, 45°, 60° and 90° (full wrap) and the resulting changes of the drum’s coal loading rate are explored, as shown in Fig. 13. In the case of ejection loading, the drum’s coal loading rate monotonically increases with the increasing wrap angle, but in a slight way. The coal loading rate under the condition of wrap angle of 30° is only 2% lower than that in the fully enclosed state. In case of wrap angle of 60°, compared with the fully enclosed state, the coal loading rates in both ejection and pushing loading modes are almost the same. The changes of drum’s coal loading rate with wrap angle of the cowl under the pushing loading are consistent with those under the ejection loading, and the coal loading rate is slightly improved when the wrap angle increases from 30° to 45°. Moreover, as the wrap angle increases from 45° to 90°, the coal loading rate does not increase significantly, only by 0.8%.

Therefore, based on the results of different wrap angles under two loading modes, a 45° wrap angle of the cowl is enough for the improvement of the coal loading rate. That is to say, the projection area of the cowl accounts for half of the diameter of the drum, rather than a full enclosed one, meaning that the projection area of the cowl fully covers the drum.

### Discussions

Based on the single-factor study on the above three factors (the offset distance, tilt angle and wrap angle of coal cowl), it can be seen that the coal cowl provides a closed space for the drum conveyor, which can significantly improve the coal loading capacity of the drum, and that the loading rate can be effectively increased by more than 20%. Nevertheless, the problems of drum chocking and recycle coal brought about by the smaller offset distance of the coal cowl cannot be ignored. Hence, it is necessary to determine a reasonable offset distance of the coal cowl after comprehensive consideration. Comparing the influence of coal cowl on the coal loading rate of the drum under different loading methods, the coal cowl offers more significant enhancement when the drum is pushed and loaded with coal. Due to the different loading mechanisms of coal flow when the drum is ejected and pushed, the influence of the tilt angle of the coal cowl on the coal loading performance of the drum varies greatly. The tilt angle of the coal cowl exerts a negative influence on the ejection loading rate of the drum, while the drum loading rate can be further improved within a certain value range in the pushing loading mode. Therefore, according to the different coal seam conditions, the loading method of the drum should be determined, and the tilted angle of the coal cowl should be adjusted to further improve the loading rate when pushing loading is adopted. Due to the relatively small influence of the wrap angle of the coal cowl on the coal loading performance of the drum under the two loading methods, a smaller wrap angle can be used in the manufacturing process of the coal cowl to meet the requirement of improving the coal loading rate of the drum as well.

## Analysis of multiple parameters and interaction effects

Based on the above results and analyses, it can be seen that the three factors of the cowl have an impact on the coal loading rate. Rational selection and configuration of the parameters matter a lot to clarifying the significance of each factor and its interaction effects to the coal loading performance of the drum, and to determining the optimal parameter selection range. The response surface method (RSM) can obtain the parameter range and matching relationship corresponding to the optimal results in the range of factor levels, and has been widely used in the field of structural optimization and parameter selection of construction machinery^40,41^. Therefore, this paper also employs the RSM to study the influence of cowl parameters and their interaction effects on the coal loading performance of the drum. The experimental simulation design of the drum and its results under both ejection and pushing loading are shown in Tables 7 and 8, respectively. Among them, according to the experimental results of single factor, the coal cowl’s offset distance is set within 0–300 mm, the tilt angle being 0–30°, and the wrap angle being 0–90°.

**Table 7:** Design scheme and results of multifactorial interaction experiment under ejection loading.

Simulation Run	*X*_1_–*a* (mm)	*X*_2_–*α* (°)	*X*_3_–*θ* (°)	*Y*_1_-loading rate (%)
1	150	15	60	73.25
2	150	0	90	81.14
3	150	0	30	79.00
4	300	15	30	70.89
5	300	15	90	69.07
6	0	0	60	83.96
7	0	15	30	73.65
8	300	30	60	66.95
9	150	30	30	69.75
10	150	30	90	74.49
11	0	30	60	73.12
12	300	0	60	76.56
13	150	15	60	72.27
14	150	15	60	74.76
15	150	15	60	71.93
16	150	15	60	75.07
17	0	15	90	82.40

**Table 8:** Design scheme and results of multifactorial interaction experiment under pushing loading.

Simulation run	*X*_1_–*a* (mm)	*X*_2_–*α* (°)	*X*_3_–*θ* (°)	*Y*_1_-loading rate (%)
1	150	15	60	75.03
2	150	0	90	72.82
3	150	0	30	70.08
4	300	15	30	69.19
5	300	15	90	72.67
6	0	0	60	77.59
7	0	15	30	69.34
8	300	30	60	68.42
9	150	30	30	68.77
10	150	30	90	71.02
11	0	30	60	74.40
12	300	0	60	70.52
13	150	15	60	73.13
14	150	15	60	74.79
15	150	15	60	74.56
16	150	15	60	72.98
17	0	15	90	76.63

### Ejection loading mode

According to the multifactor interaction experiment in Table 7, the ANOVA results under the factors and interactions corresponding to the drum’s coal loading rate are obtained, as shown in Table 9. Under the ejection loading mode of the drum, the coal cowl’s offset distance, tilt angle, and wrap angle, as well as the interaction between offset distance and wrap angle, all exert extremely significant effects on the coal loading rate. The priority order of the effects of various factors and their interactions on the drum’s ejection loading is *α* > *a* > *a·θ*>*θ*>*α·θ*>*a·α*, while the coal cowl’s offset distance and wrap angle produce the smallest effects.

**Table 9:** Variation results of coal loading for each factor under ejection mode.

Source	Mean square	F value	*P* value
*a*	109.96	67.14	< 0.01
*α*	165.17	100.84	< 0.01
*θ*	23.84	14.56	< 0.01
*a·α*	0.3782	0.2309	0.6455
*a·θ*	27.93	17.05	< 0.01
*α·θ*	1.69	1.03	0.3435

**Figure 14:**
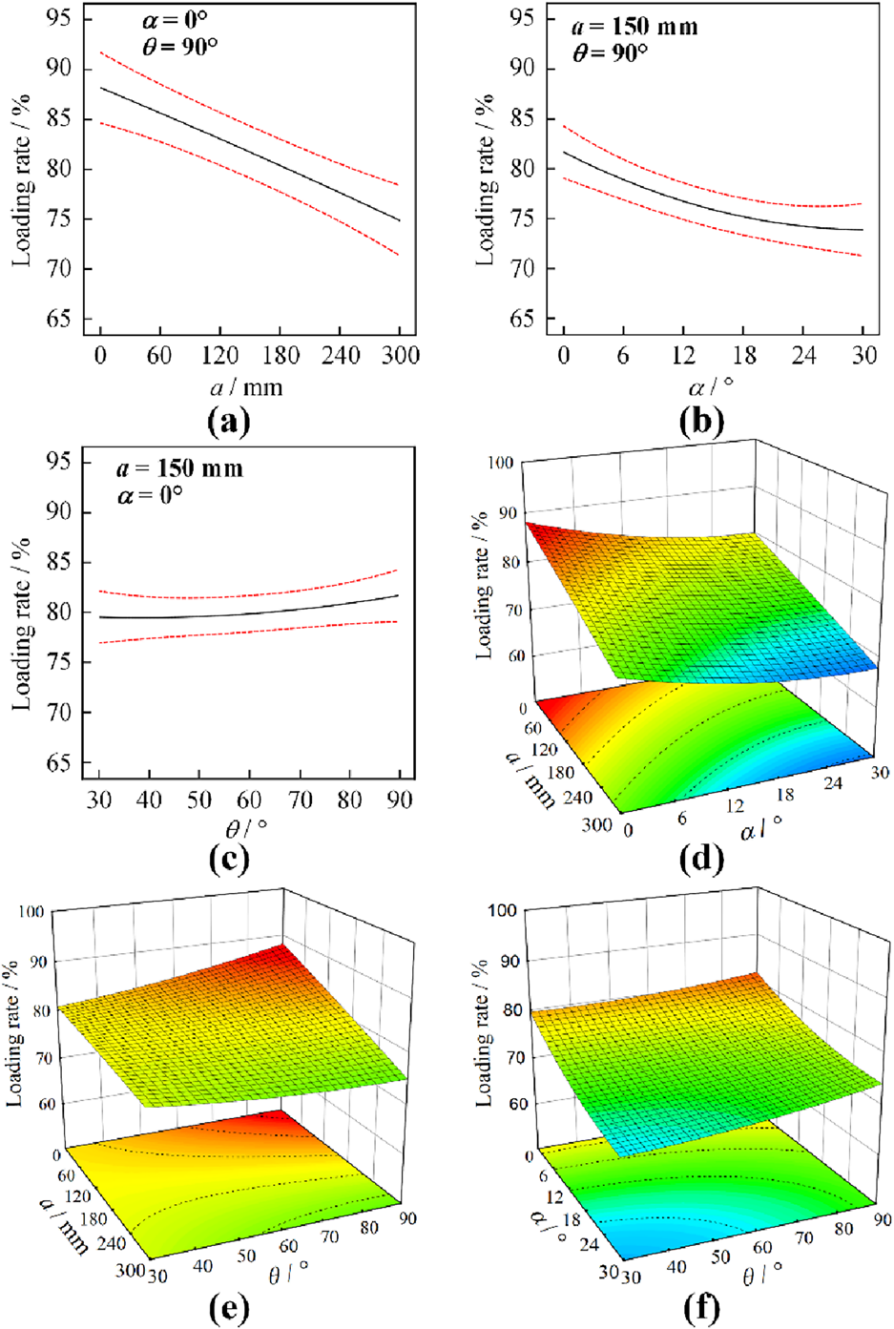
Response surface analysis results of the parameters of the cowl under ejection loading mode.

The changes of coal loading rate under ejection loading with coal cowl’s offset distance, tilt angle and wrap angle and their interactions are shown in Fig. 14. Specifically, Fig. 14a–c exhibit the results of single-factor variation, where 95% confidence interval is set between the red dashed lines; Fig. 14d–f present the results of the coal loading rate under the interaction of two factors. From Fig. 14a–c, it can be seen that under the ejection coal loading, the influence of coal cowl parameters on the drum’s coal loading rate almost always shows a monotonous change; more specifically, the coal cowl’s offset distance and tilt angle have a negative influence on the coal loading rate, respectively. In contrast, the wrap angle has a positive influence on the loading rate, which is in good agreement with the results in the single-factor study. In addition, as shown in Fig. 14d–f, the results of the interaction effects of the three factors prove that the response surfaces are relatively flat and that almost all of them obtain their peaks at the intersection of the two planar coordinate axes. As discussed above, although the smaller offset distance of the coal cowl has a better effect on the enhancement of the drum’s coal loading rate, the offset distance, if too small, will easily increase the drum’s conveying power demand, and bring about drum choking and coal over-crushing. Therefore, optimization is carried out in the offset distance of 150–225 mm, and the optimal parameters of the coal cowl, i.e., the offset distance, tilt angle, and wrap angle for predicting the optimal coal loading rate are 150 mm, 0°, and 90°, respectively, and the predicted optimal coal loading rate is 81.72%. The regression prediction equation of drum’s coal loading rate is given by Eq. (5).

5 $$\begin{aligned} Y_{1} & = f_{e} (X_{1} ,X_{2} ,X_{3} ) \\ & = 79.257 + 0.011X_{1} - 0.662X_{2} + 0.024X_{3} \\ & \quad - 0.001X_{1} X_{3} \\ \end{aligned}$$

### Pushing loading mode

Based on the interaction experiments in Table 8, the ANOVA results when the drum is pushed and loaded with coal are obtained, as shown in Table 10. Under the pushing loading mode, the influence of coal cowl’s tilt distance on the drum’s coal loading rate is extremely significant; the influence of wrap angle on the drum’s coal loading rate is significant within the 95% confidence level interval; the influence of tilt angle and the interaction between other factors are not significant. The priority order of the effect of each factor on the drum’s ejection coal loading is *a* > *θ* > *α·*>*a·θ*>*a·α*>*α·θ*, and the interaction of coal cowl’s tilt angle and wrap angle exerts the least effect on coal loading rate.

**Table 10:** Variation results of coal loading for each factor under pushing mode.

Source	Mean square	F value	*P* value
*a*	36.81	13.92	< 0.01
*α*	8.82	3.34	0.1105
*θ*	31.05	11.74	0.011*
*a·α*	0.2970	0.1123	0.7473
*a·θ*	3.63	1.37	0.2797
*α·θ*	0.06	0.0227	0.8845

*Significant at 95% confidence interval.

**Figure 15:**
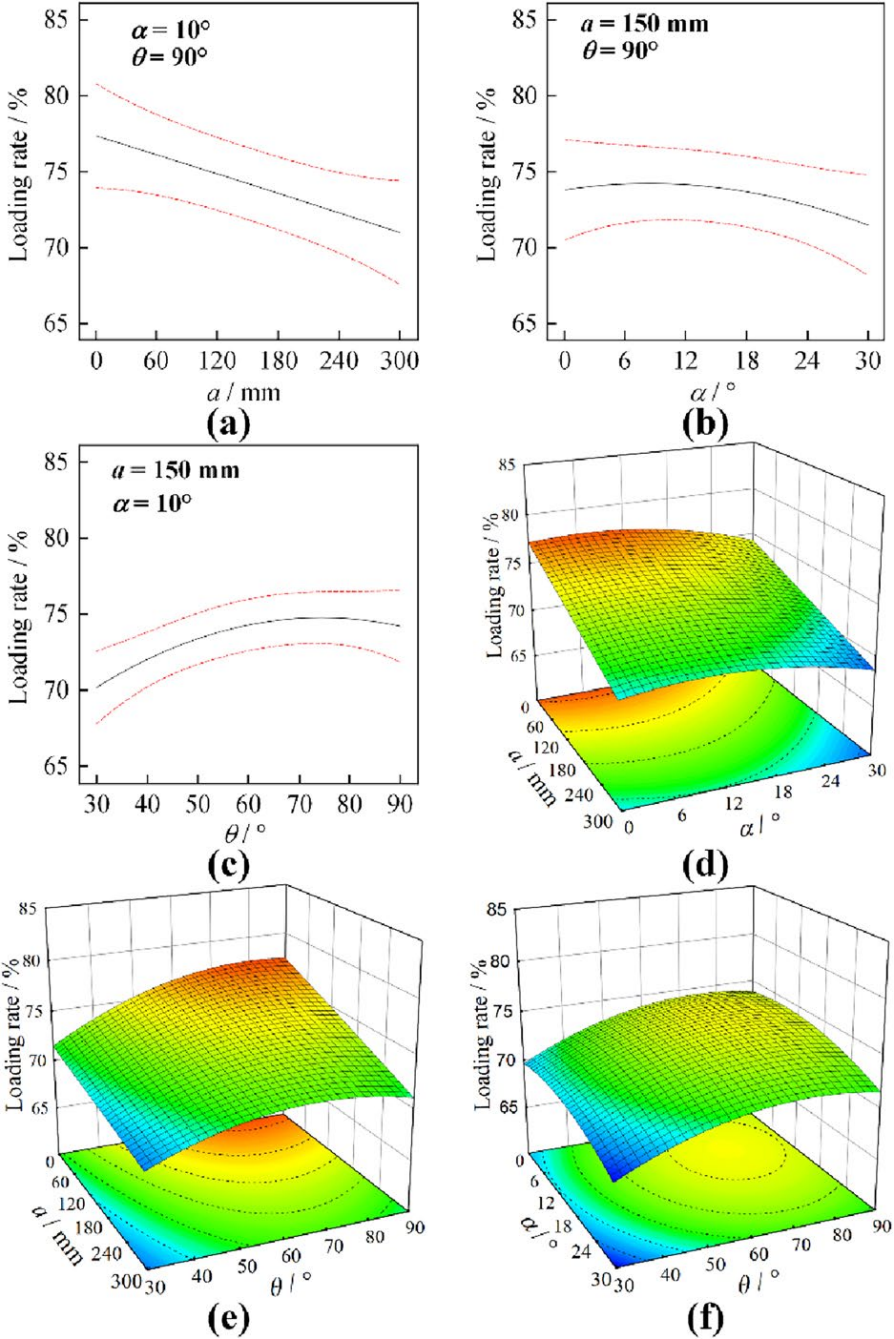
Response surface analysis results of the parameters of the cowl under pushing loading mode.

The variations of coal loading rate under drum ejection loading with coal cowl’s offset distance, tilt angle and wrap angle and their interactions are shown in Fig. 15. It can be seen from Fig. 15a that the single factor of coal cowl’s offset distance has a monotonous linear influence on the coal loading rate of drum under pushing loading, which is slightly different from the results of the single-factor experiments, but with the same trend of change as a whole. The change curve between the drum’s pushing loading rate and the coal cowl’s tilt angle is parabolic as presented in Fig. 15b, and reaches the peak value in the 12–18° interval, which are also consistent with the results of the single-factor experiment. Along with the increasing wrap angle of coal cowl, the coal loading rate of drum under pushing loading firstly rises rapidly, and then slows down and tends to stabilize, as can been in Fig. 15c. Analyzing the change rule of coal loading rate under the interaction of two factors in Fig. 15d–f, it can be known that the interaction between offset distance and tilt angle is stronger when the offset distance is smaller. The coal loading rate shows a parabolic tendency with the change of tilt angle, which is mainly due to the fact that the coal cowl deflects to the coal loading zone to provide additional axial flow velocity for the coal flow in case of a smaller tilt distance. In Fig. 15e, when the wrap angle is less than 60°, the interaction between the offset distance and the wrap angle is stronger. The coal loading rate increases with the decrease of the offset distance and the increase of the wrap angle as a linear and nonlinear function, respectively, with a steeper response surface. Obviously, in case of a smaller offset distance of the coal cowl, the smaller wrap angle is not enough for the drum, increasing the chance that the particles will be thrown into the airspace and that the coal cowl will be cast into the goaf directly. Therefore, when the wrap angle is less than 60°, the fashion coal rate increases significantly with the wrap angle. According to Fig. 15f, the response surface is flat under the interaction of tilt angle and wrap angle, which proves that the interaction of the two is not strong. Similarly, the optimization is carried out in the range of offset distance 150–225 mm, thus getting the optimal values of offset distance, tilt angle and wrap angle, namely, 150 mm, 8.715°and 74.432°, respectively. The predicted optimal coal loading rate is 74.79%. The regression prediction for the coal loading rate of roller can be expressed by Eq. (6).

6 $$\begin{aligned} Y_{1} & = f_{p} (X_{1,} X_{2} ,X_{3} ) \\ & \quad = 61.709 - 0.003X_{1} + 0.104X_{2} + 0.382X_{3} \\ \end{aligned}$$

## Conclusions

As an effective means to solve the problem of low drum loading rate of thin seam coal shearer, the auxiliary loading is affected by the installation parameters. In this paper, the model of auxiliary drum loading assisted by cowl is established with the help of discrete element method (DEM), and the coal loading process of the shearer drum with cowl is further investigated. The main conclusions are drawn below:

1. Cowl provides a better improvement effect during drum pushing loading than ejection loading. The coal loading rate of the drum decreases linearly with the increase of the offset distance of the coal cowl during drum ejection loading, while the coal loading rate decreases first and then slightly increases with the increase of the offset distance under drum pushing loading. Although a small offset distance can significantly improve the coal loading rate of the drum, the torque of the drum will also increase significantly accordingly, and the problems of drum clogging and coal circulation will become serious.
2. Under drum ejection loading, the coal loading rate linearly decreases with the increase of cowl’s tilt angle. Under drum pushing loading, the coal loading rate first increases and then decreases with the increase of cowl’s tilt angle. Under both loading modes, the coal loading rate increases with the increasing wrap angle of the coal cowl, and the coal loading rate at a 45° wrap angle is almost equal to that at a 180° wrap angle.
3. The three parameters of the cowl that affect the coal loading rate of the drum are analyzed by ANOVA, and the factors that exert the most significant influence on the coal loading rate of the drum during ejection and pushing loading are tilt angle and offset distance, respectively; Meanwhile, with the help of the response surface method, the influence of the factors and their interactions on the coal loading rate of the drum are explored, the prediction function for the rate is given, and the optimal parameter combinations of the cowl are obtained, corresponding to a 20% increase in the coal loading rate as compared to the previous prediction.

## Data Availability

The datasets used and/or analysed during the current study available from the corresponding author on reasonable request.
